# Ebola and Public Authority: Saving Loved Ones in Sierra Leone

**DOI:** 10.1080/01459740.2019.1609472

**Published:** 2019-05-20

**Authors:** Melissa Parker, Tommy Matthew Hanson, Ahmed Vandi, Lawrence Sao Babawo, Tim Allen

**Affiliations:** a Department of Global Health and Development, London School of Hygiene and Tropical Medicine, London, United Kingdom; b Department of Sociology and Social Work, Njala University, Bo, Sierra Leone; c Department of Community Health and Clinical Studies, Njala University, Bo, Sierra Leone; d Department of Nursing, Njala University, Bo, Sierra Leone; e Department of International Development, London School of Economics and Political Science, London, United Kingdom

**Keywords:** Ebola, Sierra Leone, local learning, people’s science, public authority

## Abstract

It is unclear how public authorities shaped responses to Ebola in Sierra Leone. Focusing on one village, we analyze what happened when “staff, stuff, space, and systems” were absent. Mutuality between neighbors, linked to secret societies, necessitated collective care for infected loved ones, irrespective of the risks. Practical learning was quick. Numbers recovering were reported to be higher among people treated in hidden locations, compared to those taken to Ebola Treatment Centres. Our findings challenge positive post-Ebola narratives about international aid and military deployment. A morally appropriate people’s science emerged under the radar of external scrutiny, including that of a paramount chief.

Following a visit to Liberia during the 2013–2016 Ebola epidemic, Paul Farmer commented: “Without staff, stuff, space, and systems, nothing can be done” (2014:38). He was writing at a time of widespread fear and panic that Ebola in West Africa was spiraling out of control. The most affected countries had experienced decades of armed conflict, and subsequent endeavors to re-build fragile health systems had failed (Abramowitz 2017; Bardosh, Leach and Wilkinson 2016). With insufficient medical practitioners, minimal facilities to diagnose Ebola and unreliable and limited supplies of medicines, it was easy to see why – following the initial outbreak in Gueckedou prefecture, Guinea in December 2013 – the virus was spreading so quickly. In August 2014, for example, a WHO Response Team noted that the number of cases was doubling every two to three weeks. If left unchecked, the exponential rate of increase would have a devastating impact on the region, and across the globe (WHO Response Team, cited in Kelly 2018). By September 2014, the United States Center of Disease Control (CDC) was predicting that, in the worst-case scenario, there would be around 1.4 million cases of Ebola by mid-January 2015 in West Africa alone (Meltzer et al. 2014). These terrifying predictions, in combination with pleas for assistance by leaders in global health and staff employed by INGOs (such as Médecins Sans Frontières), played a crucial role in securing the delivery of substantial medical humanitarian assistance. Indeed, more than $3.5 billion was spent responding to the epidemic. This amount was a staggering 150% more than the combined annual government budget of $2.37 billion for the three most affected countries – Guinea, Sierra Leone, and Liberia (Dubois and Wake 2015).

The scale and nature of the response was profoundly shaped by the United Nations, with the UN Security Council passing a resolution stating that: “the outbreak is undermining the stability of the most affected countries … [and] the unprecedented extent of the Ebola outbreak in Africa constitutes a threat to international peace and security” (UN Security Council 2177 2014:1). This resolution was swiftly followed by the establishment of a UN Mission for Emergency Ebola Response (UNMEER), which had the explicit intention of assisting national and bilateral agencies with the rapid roll out of care and treatment under the leadership of the WHO. Reflecting the perceived seriousness of the situation, these steps had unanimous support from all member states of the UN General Assembly.

By linking the Ebola epidemic to state instability as well as regional and global security, the UN Security Council facilitated a highly militarized approach to treatment and containment. This involved national and international armed forces, and it was strongly supported by MSF – reversing the agency’s previous emphasis on the importance of not linking humanitarian assistance with enforcement procedures (Allen 2018). Although the precise nature of military engagement varied between Guinea, Sierra Leone and Liberia, all three governments used their armies to control access to hospitals and Ebola Treatment Centres (ETCs), identify new cases, impose road blocks and assist with “lockdowns.” A further 5,000 international military personnel were deployed by China, Canada, France, Germany, UK, and the US to construct ETCs across the region (McKay and Parker 2018). Militarizing humanitarian assistance in this way provoked strong reactions, with some scholars and commentators arguing that it could end up being counter-productive (e.g. de Waal 2014; Abramowitz, Rodriguez and Arendt 2014). Others, however, took the view that it was regrettable but necessary (e.g. Dizard 2014; Farmer 2015; Walsh and Johnson 2018), and they accepted that boots on the ground were probably the only viable way of deploying biomedical “staff, stuff, space, and systems” at speed.

In the event, national and international armed forces found it hard to act as quickly as anticipated, and when they did, results were mixed (Kamradt-Scott et al. 2016). Nevertheless, the cataclysmic scenarios predicted in August and September 2014 did not occur. By the end of the epidemic in March 2016, a total of 28,616 cases and 11,310 deaths had been officially recorded (World Health Organization 2016). This raises the question: what happened in the very many locations in which “staff, stuff, space, and systems” never actually arrived, arrived too late to make much difference or were actively resisted by the population? Assuming the virus did not just burn itself out, what was done, who was responsible, and what kind of public authority influenced what occurred?

Many claims have been made about what was significant. These include effective partnerships between national/international armed forces and INGOs; improvements in biomedical therapy; increasingly effective political leadership; rolling out community care centers; contributions from paramount chiefs; and local learning. All these factors may have been important at different times, and in different places, but there is a paucity of evidence. Mindful of Murray Last’s article about “the importance of knowing about not knowing” (1981), it is vital to keep an open mind and to seek answers grounded in fine-grained, comparative research.

Anthropologists have started to engage with these issues. Wilkinson and Fairhead (2017), for example, have drawn on their long-term ethnographic knowledge of the region and activities with the Ebola Response Anthropology Platform (http://www.ebola-anthropology.net/), to reflect on the different ways in which people responded in Guinea and Sierra Leone. They noted that as the epidemic spread in Guinea, “acts of violence or everyday resistance to outbreak control measures repeatedly followed, undermining public health attempts to contain the crisis;” whereas “in Sierra Leone, defiant resistance was rarer” (2017:14). They go on to contrast the legacies of French colonial direct rule in Guinea with British indirect rule in Sierra Leone, showing how these legacies differentially shaped political responses to the epidemic.

Other anthropologists have emphasized the capacity of populations to adapt their practices, highlighting the role of local initiatives (Abramowitz et al. 2015) and local learning (Richards 2016a); and, in the case of Richards, suggested that this was an important factor in curtailing the spread of the virus. In Jawei chiefdom, Kailahun District, Sierra Leone, for example, Richards described how task forces were established, under the guidance of the paramount chief, with a view to finding cases, reducing movement between villages and imposing bylaws. These initiatives were deemed to be so helpful in containing transmission that they were subsequently replicated across the country with the support of the government (including the military) and NGOs. Additionally, Sharma and colleagues (2014) reported how the “local population” contributed to the development of a comprehensive response strategy in Lofa County, north-western Liberia; and Abramowitz and colleagues’ (2015) work in urban Monrovia, Liberia documented strategies being carried out by community leaders.

These studies are indicative of local factors that may have shaped the course of Ebola in particular places. However, it is not altogether clear what counts as “local.” In Sierra Leone, the paramount chiefs and their subordinates were by no means the only actors, and the “local” population in Liberia is undefined in the article by Sharma and colleagues (2014). Similarly, Abramowitz and colleagues (2015) did not mention if community leaders were formally appointed, and if they were, by whom. Consequently, it is not clear how they related to other kinds of authority within the area. Nevertheless, the emphasis on local initiatives and local learning is helpful. It provides a much-needed focus on what actually happened and the way in which ideas and behavior changed during the epidemic.

In this article, we build on this growing literature. We present a detailed account of an Ebola outbreak in Mathaineh village, Ribbi chiefdom, Sierra Leone. Events are analyzed with a public authority lens, with public authority being defined as “any kind of authority beyond the immediate family which commands a degree of consent” (CPAID 2018:8). The phrase “commands a degree of consent” is important, because there may be circumstances in which public authority is asserted, but does not command consent and ends up being ignored, while there may be other circumstances in which assertions are backed by such a degree of force that consent becomes irrelevant and authority reduced to an exercise of power without legitimacy. For us, public authority requires a quality of legitimacy, although by no means necessarily formal legality. That legitimacy is enhanced where it enables people to act in mutual ways, whereby they are able to treat others, in the most part, as they would want to be treated themselves.

## Public authority and Ebola in Sierra Leone

With respect to public authority during the Ebola epidemic in Sierra Leone, Richards (2016a) and Wilkinson and Fairhead (2017) suggest that, in practice, paramount chiefs were often more influential than conventional forms of official governance, international agencies or military forces. Richards also argues that the governmental spaces that the chiefs established allowed “a people’s science” to develop. He initially used the term “a people’s science” in his seminal book on agriculture (Richards 1985), which showed that farmers are continuously experimenting and learning in Sierra Leone, to ensure optimal yields. It was a key aspect of his argument that the knowledge of international or national agricultural experts was often flawed in comparison to what farmers knew themselves. Other scholars, inspired by this insight, carried out related studies on agriculture elsewhere (e.g. Fairhead and Leach 1996). Their work has proved influential in changing the way in which African agricultural practices are understood, but a caveat was introduced in a much-read book by Robert Chambers (1997). Chambers noted that while farmers experiment with crops, they rarely do so with their children and loved ones. Human relationships tend to be grounded in spiritual and moral concerns, and social relationships are perceived to be so important that they are not treated like “things” to be tested. With respect to Ebola, however, the containment of the population, and the experience of seeing the effects of the virus, made learning quick, and paramount chiefs were not the only ones who did so.

It should be emphasized that paramount chiefs, and the chiefly system associated with them, is an introduced hybrid of customary and formal institutions, dating back to the time of the Protectorate. British colonial officials created administrative arrangements for indirect rule beyond Freetown and its coastal hinterlands. Paramount chiefs were appointed from a small pool of elite families, and they were expected to exercise sole authority over local government, with mentoring and support from British officials. This subsequently evolved into a system of electing paramount chiefs, with candidates normally holding the position for life, unless they were removed by colonial authorities. Following independence in 1961, they became accountable to the national government in a similar way, and continued to have considerable, externally enhanced, authority in much of the country. During the war in the 1990s, and subsequent years of political change, their authority was pervasively undermined, even after the UK controversially supported their restoration in the post-war period. However, the Ebola epidemic had the effect of re-invigorating the chieftainship system. National and international Ebola responses worked in collaboration with paramount chiefs, often in ways that replicated strategies adopted under British protectorate rule. Unsurprisingly, expanding their remit in this way was not always experienced as benign, and was resisted in some locations by those who questioned their legitimacy to intervene in intimate spaces of daily life. This was the case in Ribbi chiefdom where the Temne speaking population has a history of anti-chief activity.

At the time of the epidemic, there were 149 chiefdoms across Sierra Leone. Each had, at least ostensibly, a clear hierarchy with the paramount chief being the highest formally recognized form of customary authority, followed by the speaker, section chief, and town [village] chief. It is important to appreciate that while the terms “chief” and “chiefdoms” evoke a sense of tradition and locality, many of these chiefs were anything but “traditional.” Some paramount chiefs were born and/or educated in the US or Europe, some had dual citizenship and, even if their primary residence is now Sierra Leone, they often have relatives living and working overseas. A paramount chief in Moyamba district, for example, has a wife living in the US, and he left Sierra Leone during the epidemic to visit her and to receive medical treatment. Such arrangements are not uncommon. Also, in quite a few locations, paramount chiefs work closely with multinational companies, with some openly involved in diamond mining (Fanthorpe and Maconachie 2010). Others were political appointments made after the war and linked to the ruling party’s strategic interests. How all this related to the practice of public authority in particular places inevitably varied. In some areas, there were clearly tensions. Honigsbaum (2017), for example, noted in passing that there were several cases of “village headmen” in Western District and Port Loko who rejected official regulations designed to contain Ebola and did not report illnesses and deaths, presumably to more senior chiefdom authorities and other officials involved in the response. There were also other institutions which could challenge or bypass their remit, notably different kinds of “secret societies.” These societies are prevalent across Sierra Leone (Albrecht 2016; Dorijahn 1959; Fairhead 2014; Little 1949; Richards 2016b). Both women and men are members of societies, with each having their own hierarchies, rituals, and rules. The prevalence of secret societies is integral to social bonding and requires that people collectively keep many things to themselves. Those hidden things may reveal the lived realities of public authority more than anything else, as they do in Mathaineh (where initiation ceremonies were openly occurring during fieldwork).

## Field site and methods

Mathaineh is located in Ribbi chiefdom, one of 14 chiefdoms in Moyamba district, Southern Province. At the time of fieldwork, the village had a population of 272. Residents are Muslims and speak Temne. Some also speak Krio. In common with other villages in the district, they rely primarily on subsistence agriculture. Ribbi chiefdom borders Port Loko and Western Area Rural, and there are 28 crossing points to these districts. People and products move back and forth, and many people are closely connected to relatives living in the two districts as well as the capital city, Freetown. The chiefdom headquarters in Bradford is 15 km from Mathaineh and 90 km from Freetown, and the district headquarters in Moyamba town is 112 km from the city.

Research in Ribbi chiefdom, and other parts of Moyamba district, occurred between March 2017 and March 2018. Initial enquiries established that the military had intervened in various locations, including Mathaineh, following reports of “secret burials.” To further understand these events, fieldwork was carried out in the village. This involved participatory methods, open-ended, unstructured interviews and informal group discussions. Every effort was made to spend time with a wide range of people including farmers, birth attendants, local healers, teachers, village elders, imams, Ebola survivors. Fieldwork in Mathaineh was supplemented and contextualized by 30 interviews with officials working for the Ministry of Health and Sanitation (MoHS) at a district and national level. These included disease surveillance officers, community health workers, nurses, and midwives. Additionally, 26 interviews were undertaken with chiefdom authorities, notably paramount chiefs, section chiefs, chiefdom speakers, town [village] chiefs, and people working on the Ebola response for the Sierra Leonean army, police, and INGOs. The latter included people who were employed to carry out “safe and dignified” burials within the district. Interviews were usually tape recorded and subsequently translated from Krio, Mende or Temne into English.

The first confirmed case of Ebola at Moyamba hospital was on September 3, 2014. Staff ran away in terror. The patient was taken to a holding center on the outskirts of the town. This was a dilapidated building with few windows for ventilation, no running water or sanitation facilities. People suspected of having Ebola were sent to the holding center, before being passed on to an ETC in Kenema or Kailahun. As the cases mounted, international agencies worked closely with staff from the MoHS, Ministry of Defence and district council to build an ETC in Moyamba town. The center reportedly opened in December 2014, with funding and technical assistance from a range of donors including Medicos del Mundo, World Vision, Save the Children Fund, Action Aid, Moyamba Dependents Association, World Food Programme, and the WHO.

The District Ebola Response Centre (DERC) was established shortly beforehand. It was part of the newly-designed, command-and-control response architecture, accountable to the National Ebola Response Centre (NERC), and it played a pivotal role in organizing the response. In particular, the DERC co-ordinated activities between the District Administrative Council, chiefdom authorities, INGOs, and the Sierra Leonean military and police. They focused attention on the following pillars: quarantine, surveillance, burials, case management, social mobilization, diagnosis, and alerts. However, these district-level strategies were largely unknown or perceived as irrelevant in Mathaineh.

Indeed, village elders spoke with evident frustration about the way in which resources by-passed the village. A government-funded, primary school was meant to have been located in the village in 2011, but ended up being constructed in a place that was too far away for their children to walk to easily; and they resorted to building their own school and appointing volunteer teachers. Similarly, a maternal and child health post (MCHP) was established in 2012 but was located a couple of kilometers away in a neighboring village. Records indicated that women and children from Mathaineh attended the MCHP, but there were few perceived benefits. Although childhood vaccinations, growth monitoring, nutritional supplements, bednets for the prevention of malaria, and antenatal care is provided at the MCHP free of charge, there was little else on offer. Antibiotics and paracetamol, when available, had to be paid for. The nearest community health center (CHC) is located in Bradford. This center stocks medicines for a broader range of conditions and offers diagnostic tests for infections such as HIV, TB, malaria, and dysentery. Staff are also trained in the clinical management of conditions such as typhoid, measles, pertussis, and complications arising from malaria. However, residents from Mathaineh rarely sought care at this center, due to a combination of travel costs, the charge for services, and “dismissive attitudes” of staff. When Ebola broke out, it was no surprise to any of them that no-one in the chiefdom or district administration asked for a village contact tracer to be identified, even though it was government policy for every village to have one.

## Ebola in Mathaineh

The outbreak began when a young man returned to his home village, Mathaineh, from Waterloo (an urban area close to Freetown) with his heavily pregnant wife. Within days he developed signs of Ebola infection: diarrhoea, vomiting, bleeding from orifices. His sickness was not reported. Instead, he was cared for by his relatives in the family home. When he died, he was washed and wrapped in white cloth in accordance with local practice; and a decision was swiftly made not to inform chieftain and district authorities in Moyamba about his death. Instead, he was buried close to his house. His wife gave birth in the village a few days after his death, with the infant delivered by several “traditional birth attendants,” who were members of Mathaineh’s female secret society. Unfortunately, the wife went on to develop identical signs and symptoms as her husband, and tragically died from Ebola a few days later. This was followed by the death of her newborn infant, and one of the birth attendants also fell ill.

None of the deaths were reported to the chiefdom authorities; and no attempt was made to ring 117, the nationwide emergency number (established by the MoHS), about the birth attendant’s illness. Instead, a meeting was held in the village to discuss what to do. The town (i.e. village) chief, reportedly reflecting the views of the village’s secret societies, declared that in the event of anyone else falling ill, s/he should be taken to the bush and cared for by a relative, if necessary, in a rapidly assembled straw hut. Such a strategy aimed to separate the ill from the well and to prevent the spread of infection. Those who fell ill were instructed to consume small amounts of pepper soup, and a drink of lime and honey. Several diseases, which were known to spread easily and quickly from one person to another, had been successfully treated this way in the past, including cholera and smallpox. To ensure this approach was adhered to, the chief went from house to house every other day to ask if anyone else had fallen ill and, if any cases were reported, to ensure they were quickly taken to the bush. People were told that if they ignored or delayed this instruction, then he would ring 117 – the implication being that they would be taken away to die with strangers. In the event of further deaths, it was also agreed that none would be reported to the chiefdom authorities. and that burials would continue to occur in the village, albeit “secretly.” Aware of a bylaw which required citizens to report all kinds of sickness and death to the chiefdom authorities – irrespective of the cause – and cognisant of the fact that anyone found not reporting a death would be fined 500,000 Leones, villagers agreed that anyone who leaked information about burials occurring in the village would be fined the equivalent sum of 500,000 Leones. Everyone agreed to be vigilant and to alert their relatives and neighbors to any strangers passing through the village, and children were told in no uncertain words not to speak to anyone about events in the village.

Over the following weeks, more and more people fell ill. Making their own empirical observations about how Ebola spread from one person to another, they refined their strategies for caring for the sick, mourning and burying the dead. One young man, for example, described how he had given a piggyback to help one of the birth attendants cross a small river. The woman was being taken to the bush as she was developing the same signs and symptoms of illness as the recently deceased woman she had assisted during birth. He had not been wearing a T-shirt at the time, and he was aware that a small amount of spittle fell from her mouth onto his back. Within a week, he fell ill with identical symptoms; and it became apparent that skin on skin contact should be avoided at all costs. This led to relatives making a real effort to avoid physical contact by placing a mixture of lime and honey, water and/or pepper soup in the reach of people who were sick, but ensuring they did not touch them.

**Figure 1.**
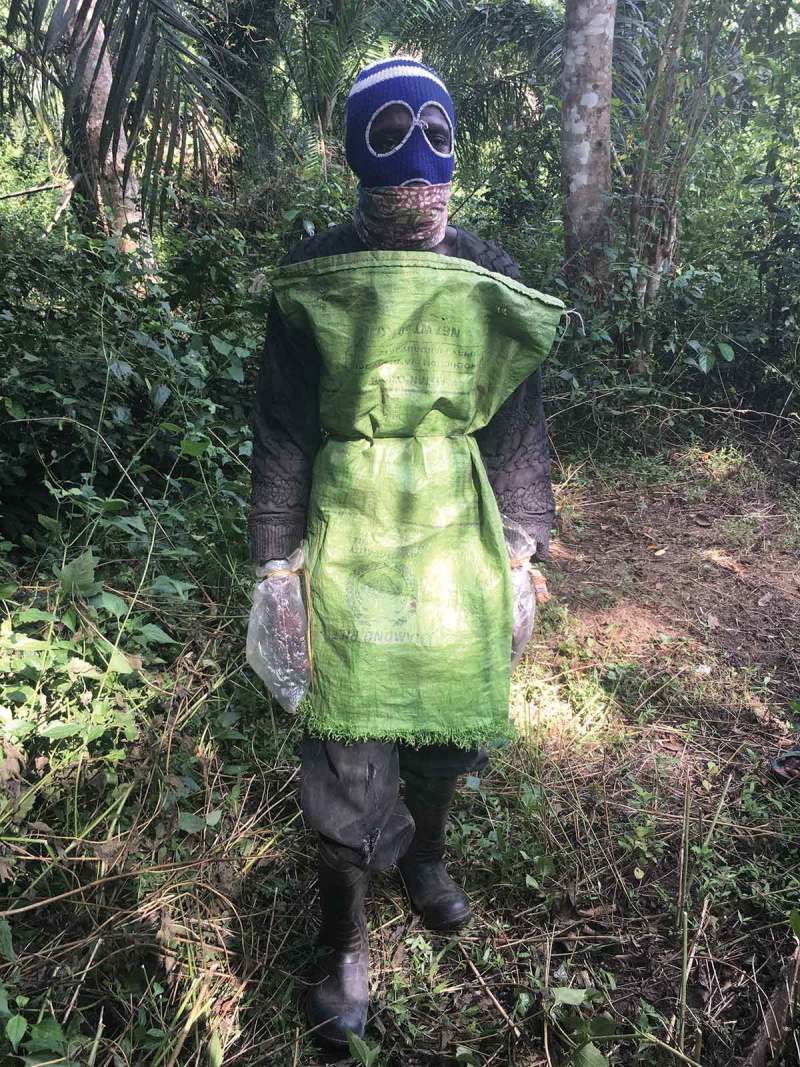
Member of village burial team, wearing locally made personal protective equipment.

Not all people survived. An *ad hoc* village burial team involving a group of 13 young men emerged. Although longing to bury their kin in respectful ways, they quickly learnt to refine their practices. Bodies were no longer washed or publicly buried. Instead, they developed their own home-made personal protective equipment (PPE). As indicated in Figure 1, they found out what would protect them and used plastic bags for gloves, sugar or rice sacks to wrap around their body, wellington boots to cover their feet, and woolen facemasks (which otherwise might have been used to protect themselves from dust on motorbikes). They never used the same equipment twice. Indeed, they buried the plastic bags and sacks with the body to ensure there was no cross infection. Following a burial, they also washed their hands and feet with soap and water, and boiled their boots in lime.

Considerable discipline was shown by those doing the burials. *Recces* were deployed to walk around the village and alert the team if any stranger was seen entering the village. On several occasions, outsiders became suspicious, and great lengths were taken to put them off their tracks. The nurse based at the MCHP in the neighboring village, for example, had been informed that the index case was ill. When she followed it up and asked to see him, she was told that he had returned to Freetown. Suspicious, she asked to talk to him on the phone. Villagers contacted the deceased man’s daughter and asked her to pretend to be his wife when the nurse rang the number. She duly did this, saying he was fit and well but unavailable to speak. This threw the nurse off the scent, at least for a while. However, with cross-infection continuing, it proved impossible to keep the secret. Both the chiefdom authorities and staff employed by the MoHS ended up being alerted to events unfolding in the village.

### Militarizing the response

All these events were happening at a time of widespread fear among national and international actors. The President of Sierra Leone had made it clear that he would depose paramount chiefs if they did not assert their authority by preventing movement, ensuring the rapid reporting of sickness and the safe disposal of bodies by officially trained burial teams in PPE. In Moyamba district, the ETC had recently opened and a large number of staff from INGOs and the MoHS were working closely with senior chiefdom authorities to implement the national strategy. With increasing numbers of cases being reported in Ribbi chiefdom, and the paramount chief under intense pressure to act, officials from both the DERC and the chiefdom discussed a strategy for intervening. Describing the people of Mathaineh as “hard to deal with,” “difficult,” “lawless” and “unwilling to accept orders,” there was – they felt – little choice but to intervene with the assistance of the Sierra Leonean military.

At the beginning of December 2014, they went to the village with a group of soldiers. On arrival, they ordered people to come out of the houses and threatened to beat them, if they did not reveal where bodies had been buried. While some people fled to the bush, others stayed and remained silent. Women described how they were kicked down the main path running through the village, men described how they were hit with the butts of guns (with some still visibly suffering from injuries), and children described how they were offered large sums of money if they showed them the places where people had been buried. Eventually, a young man who did not “wish to die for Ebola” revealed a burial site. One official, reflecting on events, said that he told the staff of an international agency: “You close your eyes. You just forget about what I am going to do. I am going to use some force in this community … I called on the military guys … and they went round the village … gathered [people up] and said if you don’t tell us the facts, we will flog you.” On another occasion, a member of an official burial team described how they had been given some money “to motivate them” to “use the radical way.” In other words, senior figures within the chiefdom gave them the authority to act on their behalf and, alongside the military, to behave violently. Following the discovery of “secret” burial sites, the paramount chief suspended the section chief for neglecting his responsibilities, and the town chief for defying the bylaws.

Following intervention by the military, other cases of suspected Ebola were identified in the village. In line with government policy, these people were sent in an ambulance to the ETC in Moyamba. One woman tearfully recounted how her sick husband had reluctantly agreed to be treated at the unit. He did not survive and, in common with others who died from Ebola, his body was not returned. Her house was quarantined, with soldiers permanently located outside for 21 days. During this period, her son fell ill with identical symptoms as her husband. She did not reveal his illness to the soldiers, choosing instead to care for him with lime and honey. Remarkably, he survived.

To our knowledge, this was the only endeavor to resist government strategies of treatment and containment after the military intervention. Eight people were sent to the ETC in Moyamba, four of whom survived. Although several other deaths also occurred in the village in January 2015, they were all reported to the official burial team in Bradford. The official protocol had changed by this time. Burial teams were not only allowed to bury the deceased in places close to the homesteads, but they also allowed villagers to watch the burials and to pray near the graveside.

### Mortality and morbidity from suspected Ebola

By the end of the outbreak in January 2015, 57 cases of suspected Ebola had occurred in the village. Twenty-six of these cases occurred among women and 16 among men. A further four cases concerned children aged six to 17 years, and 11 cases occurred among children aged five years and less. Of these cases, three women, four men and one child under the age of five were treated at the ETC in Moyamba town. The recovery and fatality figures were as follows: two women and two men recovered; and one woman, two men, and one child died at the ETC. All the other cases occurred in the village, of whom 23 were women, 12 men, and 14 children (with 10 being five years or less and four aged 6–17 years). The majority (39) were taken to the bush, if Ebola was suspected. Twenty seven people recovered (16 women, 6 men, 4 children aged 6–17 years, one child under the age of five); twelve died (three women, four men and five children aged 5 or less). Recovery from suspected Ebola was reported to be higher among those people treated by relatives in the bush (27/39), compared with those receiving treatment at the district ETC in Moyamba (4/8). These data differ from official reports. Elston and colleagues (2015), for example, state there were 58 confirmed cases of Ebola up to the middle of February 2015 in the whole of Ribbi chiefdom; and Haaskjold and colleagues (2016) reported 31 cases of Ebola at Moyamba ETC, 18 of whom died. The challenges of interpreting these data are discussed in the next section.

## Discussion

### Narrating Ebola

In describing the outbreak of Ebola in this article, we have foregrounded the thoughts and experiences of residents in Mathaineh village. The details emerged during wide-ranging conversations in groups, and individually, with village elders, farmers, herbalists, traditional birth attendants, imams, current and past town (village) chiefs, and young men unofficially assisting with burials. Where appropriate, time was also taken to visit the graves of those who had been buried by the village burial team and to discuss with relatives of the deceased the events leading up to and following their death. While the deaths of those buried close to the village cannot be officially confirmed as Ebola deaths, and it is possible that some people died from malaria or other infectious diseases, relatives reported similar signs and symptoms to those of the index case; and the sequence of events suggests that they were probably infected with Ebola. In all cases, they were buried on the assumption that they had died from Ebola.

Unsurprisingly, the way in which residents spoke about the outbreak changed over time. They were acutely aware of the fact that they had flouted chiefdom bylaws, and some elders feared retribution. Consequently, there was an initial tendency to skate over the details of events and to focus discussion on the problems with the official response. In particular, early health messaging linking Ebola to the consumption of bats and monkeys left many people thinking that the disease would not come to the village as these animals were not part of their lives. Official communication suggesting that there was no effective treatment for Ebola, but that citizens should send their relatives to hospital or an ETC, was profoundly troubling too. Villagers were more than aware that across the country, the bodies of the deceased were not being returned.

With time, a more detailed account of events in Mathaineh emerged. Reflecting on the loss of life, some people spoke openly about the complex issues they faced. The suspended town [village] chief, for example, described a situation in which he had attended a meeting at the chiefdom’s section headquarters in November 2014. In the course of the meeting, town chiefs were reminded of the importance of reporting sickness and death to chiefdom authorities, and that a 500,000 Leones fine would follow if this did not happen. At the time of the meeting, his father was seriously ill, and by the time he returned home, he had died. He felt conflicted and almost rang 117, but the prospect of receiving such a large fine was a deterrent, especially as the body had already been buried and the village burial team had acted in good faith.

For all the complexity, it is clear that the people of Mathaineh were drawing on strategies which had been used to contain and treat outbreaks in the past, and were fast to learn about the new one. They separated the sick from the well, and encouraged those struggling with infection to drink a mixture of lime and honey at regular intervals. As the outbreak unfolded, strategies were revised in the light of their own empirical observations about Ebola transmission. Care was given by one, rather than several, relatives; and physical contact was avoided wherever possible. An unofficial, untrained, village-based burial team developed its own PPE from local materials to prevent skin on skin contact with the dead. Team members also adapted their burial practices, ensuring that the deceased were no longer washed and that the equipment they used to bury their dead was never used twice. This was a morally appropriate people’s science in practice.

These responses were not happening in isolation. Ebola was regularly discussed on the radio. Alarming messages were often reiterated by friends and fellow members of secret societies living in Freetown and other urban locations. A fear that ETCs were little more than places to die, an awareness that the bodies of the deceased were not being returned, and a fear that quarantine would bring economic ruin, contributed to widespread refusal to comply with the bylaws being enacted. Crucially, people in Mathaineh had no reason to suppose that official authorities responsible for implementing the national strategy had their interests at heart.

On the contrary, the outbreak of Ebola exacerbated pre-existing tensions with senior chiefdom authorities, and there was a strong sense that “Ebola money” was passing them by. The fraught situation was not helped by the close links that were perceived to exist between senior chiefdom authorities, the DERC, and the military. Further, the violence exhibited by the military reinforced a sense of public mutuality within the village. Responding appropriately to the death of a loved one is a hugely important aspect of being part of a community. Setting this aside violated moral norms that make life meaningful, and was collectively resisted. At a public meeting in January 2018, residents were openly proud of what they had done, despite the punishments incurred (Parker and Allen 2018).

### Wider implications for public health policy and practice

There is very little information about whether other villages had comparable experiences during the epidemic. However, “secret” burials are reported to have occurred in several other villages in Ribbi chiefdom, in other chiefdoms in Moyamba district, and in other districts in the southern region more generally (see, for example, News24 2014). Moreover, the “strategies of concealment” described by Ferme (2001) among the Mende in south-eastern Sierra Leone resonate in some respects with the Temne in Mathaineh village. Writing before the outbreak, Ferme argued that disguise, deferment, and ambivalence were “the product of a violent history – reflecting regional and global forces” (2001:7). Drawing on fieldwork carried out in Freetown during the Ebola outbreak, Lipton (2017) developed her argument by suggesting there was a certain amount of cooperation between the police, council members and the public to orchestrate “secret” burials. This was not the case in Mathaineh.

In some respects, events in Mathaineh echo those described by Goguen and Bolten (2017) in a place referred to as Mabele. Here, the chief (presumably the “town chief”) cooperated in secret burials but appears to have been exposed by a neighboring chief, who imposed a quarantine and triggered an aggressive intervention in Mabale by the military. It is not clear from this account whether the way in which secret burials occurred were similar to those in Mathaineh, and no mention is made of the kinds of therapy offered to the afflicted. Nevertheless, the authors indicate that efforts at avoiding surveillance and caring for loved ones were far from unique.

In addition, Goguen and Bolten (2017) do not report violence in Mabele towards those attempting to impose controls on behavior. That is a striking aspect of responses in Mathaineh too, and contrasts with an attempt to ambush and kill the paramount chief of Rotifunk in August 2014 (interview with the chair of the national council of paramount chiefs); accounts of throwing stones at ambulances and burial teams attempting to collect patients and bodies in Kailahun (Mark 2014) and Gbangbatoke, Moyamba district (interview with a member of staff at Gbangbatoke’s peripheral health unit); and vociferous protests by young men angered at the idea of taking blood from a woman thought to be infected with Ebola in Koidu, Kono district (Ruble 2014). What prompted such forms of resistance in some locations and not others are largely a matter of speculation, but it seems likely that the specificities of public authority in particular locations was the key factor.

Perhaps the most striking finding from Mathaineh is that the number of people recovering from Ebola were reported to be higher in the bush than the ETC. It does not follow that resources should no longer be allocated to future epidemics by national and/or international actors, especially as these reports are based on the perceptions and experiences of villagers, rather than biomedical testing. It does, however, suggest the need for rethinking the way in which assistance is provided and communicated. Three issues stand out: the role of home care, the approach to burials, and the importance of engaging with locally specific kinds of public authority. With respect to home care, the findings presented in this article corroborate previous suggestions made by the Ebola Response Anthropology Platform (Martineau, Wilkinson and Parker 2017) and Hewlett and Hewlett (2008) – namely that people will continue to care for relatives and friends with Ebola at home, particularly in places where the provision of biomedical health care is, at best, limited, and where the response system is slow and weak. There is, as other scholars have pointed out, a moral imperative to care for the sick (e.g. Abramowitz et al. 2015; Chandler et al. 2015; Park and Akello 2017). In these contexts, it is unhelpful to support care at ETCs and Community Care Centers at the expense of “home care.” If chlorine, buckets, gloves, boots, and PPE had been made widely available at the outset, it is likely that many more lives would have been saved, although we shall never be able to gauge the number. These points were recognized by CDC, which eventually provided guidelines in 2015 about caring for the sick while waiting for an ambulance to arrive.

With respect to burials, the data we have presented confirms that the initial protocol designed by the World Health Organization in Geneva on safe burials (World Health Organization 2014) lacked dignity and respect. From a biomedical perspective, there is no doubt that washing a dead body prior to burial is an “unsafe” practice. The person washing the body is exposed to the Ebola virus and risks spreading the virus to others. In an endeavor to halt such practices, the government of Sierra Leone made the washing of dead bodies illegal, stating that people continuing with such a practice would be imprisoned for up to two years. The events described in Mathaineh, and elsewhere, illustrate that without addressing the social and spiritual dimensions of illness and death, such a policy was always going to be misguided and counterproductive. Reflecting on events following the arrival of the military, the people of Mathaineh reminded us of their experiences with Moyamba’s ETC. They pointed out that four people from their village had died at the center. They had not been allowed to visit them while they were receiving treatment, nor had they been able to see for themselves that the people being treated had indeed died. Related to this, and in common with national policy, none of the bodies were returned to the village for burial. As a result, relatives did not know what happened to the bodies, and whether or not they were even buried. For those who died in the bush, they could at least rest in the knowledge that their graves were nearby. In the words of one village elder, “we thank God that we can identify our family members.” The significance of these issues resonates across chiefdoms and districts in Sierra Leone (e.g. Fairhead 2014; Frankurter 2016; Goguen and Bolten 2017; Lipton 2017; Richards 2016a).

Beyond the issues of home care and safe burials, our findings foreground the importance of engaging with the historical, sociopolitical and economic context in which an epidemic occurs; and the multiple, locally specific ways in which different kinds of public authority influence transmission. This is as true for Sierra Leone as it is for other parts of West Africa, but it is not the lesson that has been learnt. Instead, complex local realities are subsumed into questionable post-Ebola narratives about the achievements of international aid against the odds, or a military victory over the epidemic – see, for example, Ross and colleagues (2017) and House of Commons (2016).

The point is underlined by current developments in the Democratic Republic of Congo (DRC). Here, emphasis is being given to the rapid construction of tented treatment centers to provide biomedical therapy, and the use of a recently developed vaccine for those at risk of Ebola infection. The strategy is running into difficulties in North Kivu and Ituri provinces. By March 12, 2019, there were 923 confirmed cases, including 583 fatalities (WHO 2019). In spite of early reporting by the Congolese government, and the rapid provision of assistance by humanitarian agencies, the number of new, confirmed cases is rising. Explanations for this state of affairs include the fact that conflict and insecurity characterize day to day life in the two provinces, and frontline health workers regularly have to suspend activities for fear of being attacked. The situation is complicated by the fact that there are multiple militia groups attempting to assert authority, and it is proving difficult to counter the political narratives they have created of Ebola as a “weapon of war” and a form of “medical terrorism.” While not directly equating secret societies in Sierra Leone with militia groups in DRC, our work highlights the point that, if disease control is really the priority, then understanding and working with locally acceptable forms of public authority is essential. Rapidly responding to Ebola necessitates identifying and working with those institutions that have an established legitimacy in intimate spaces, and that allow for the mutual care of loved ones.

## Conclusion

In this article, we have analyzed an outbreak of Ebola in Mathaineh village, Ribbi chiefdom, Moyamba district, Sierra Leone, where there was a notable lack of biomedical “staff, stuff, space and systems.” By reflecting on relationships between formal, hybrid and informal authorities (such as secret societies), it has been possible to broaden the focus from initiatives promoted by paramount chiefs to activities occurring under their radar. In Mathaineh, a morally appropriate form of people’s science emerged in a context where paramount chiefs lacked accepted authority to impose externally monitored restrictions. Eventually, the army arrived to try and impose restrictions, but the precise combination of factors which brought the outbreak to a close is unknown.

Focusing on the specificities of one place is important. Much has been written about the failure of governments, UN agencies and humanitarian agencies to respond quickly and effectively to the West African Ebola outbreak (e.g. Moon et al. 2015, National Academy of Medicine 2016; World Health Organization 2015). To different degrees, the assessments emphasize the need to work with and learn from “communities,” whilst simultaneously strengthening national disease surveillance mechanisms in particular, and health systems in general. They all call for greater international coordination and leadership, and argue the case for WHO to play a pivotal role in overseeing preparedness and response activities in the future. More specifically, they emphasize the need to refine international health regulations; to focus on enhancing accountability mechanisms; to produce and share epidemiological, genomic and clinical data during epidemics; and to accelerate research and development for medicines and vaccines. In all cases, technical solutions are foregrounded, and the case is made for more and better “staff, stuff, space, and systems.”

Doubtless that would be helpful, but the impact will be limited without understanding and respecting the local norms, social values, practical capacities and public authority of affected populations. Just about everyone pays lip-service to this point, but there is a need to go well beyond vaguely evoking the notion of community engagement (Wilkinson et al. 2017). Anthropologists contributed to redirecting and refining policy during the epidemic, because they had prior knowledge about affected people. Assessing what actually occurred on the ground between 2013 and 2016 in West Africa requires comparable fine-grained assessments. As the case of Mathaineh demonstrates, it necessitates being open to surprises.
